# Current Status and Challenges of Oncolytic Virotherapy for the Treatment of Glioblastoma

**DOI:** 10.3390/ph16060793

**Published:** 2023-05-26

**Authors:** Mason J. Webb, Ugur Sener, Richard G. Vile

**Affiliations:** 1 Department of Hematology, Mayo Clinic, 200 1st Street SW, Rochester, MN 55905, USA; 2 Department of Medical Oncology, Mayo Clinic, 200 1st Street SW, Rochester, MN 55905, USA; sener.ugur@mayo.edu; 3 Department of Neurology, Mayo Clinic, 200 1st Street SW, Rochester, MN 55905, USA; 4 Department of Molecular Medicine, Mayo Clinic, 200 1st Street SW, Rochester, MN 55905, USA; vile.richard@mayo.edu

**Keywords:** glioblastoma, oncolytic virotherapy, clinical trials

## Abstract

Despite decades of research and numerous clinical trials, the prognosis of patients diagnosed with glioblastoma (GBM) remains dire with median observed survival at 8 months. There is a critical need for novel treatments for GBM, which is the most common malignant primary brain tumor. Major advances in cancer therapeutics such as immune checkpoint inhibitors and chimeric antigen receptor (CAR) T-cell therapy have not yet led to improved outcomes for GBM. Conventional therapy of surgery followed by chemoradiation with or without tumor treating fields remains the standard of care. One of the many approaches to GBM therapy currently being explored is viral therapies. These typically work by selectively lysing target neoplastic cells, called oncolysis, or by the targeted delivery of a therapeutic transgene via a viral vector. In this review, we discuss the underlying mechanisms of action and describe both recent and current human clinical trials using these viruses with an emphasis on promising viral therapeutics that may ultimately break the field’s current stagnant paradigm.

## 1. Introduction

Glioblastoma (GBM) is the most commonly occurring malignant brain tumor, accounting for 14.2% of all central nervous system (CNS) tumors and 50.1% of malignant CNS tumors in the United States [1]. Median observed survival reported by the Central Brain Tumor Registry of the United States (CBTRUS) is 8 months and accounted for 129,570 cases between 2001–2018 [1]. Current standard of care for GBM is maximal safe surgical resection, followed by radiation therapy with concurrent and adjuvant temozolomide (TMZ) chemotherapy [2]. The addition of tumor-treating fields (TTF) to standard of care is an option, associated with a median progression-free survival benefit (PFS) of 2.7 months and a median overall survival (OS) benefit of 4.9 months over adjuvant TMZ alone [3]. However, despite multimodality treatment, recurrence is universal. Treatment of recurrent GBM is not standardized. Though systemic therapies such as bevacizumab and lomustine are frequently administered, to date, no intervention for recurrent GBM has been associated with a clear survival benefit in a large-scale clinical trial [4]. Due to a paucity of effective treatments, National Comprehensive Cancer Network (NCCN) guidelines recommend all patients to be considered for clinical trial enrollment [5].

Current clinical trials for glioblastoma are varied, investigating novel drug delivery strategies, combination systemic therapy approaches, molecularly targeted therapies, and device-based treatments [3,6]. Immunotherapy approaches such as the use of immune checkpoint inhibitors, cytokine-based therapies, therapeutic vaccination, and T-cell therapies have been the focus of intense investigation [7,8]. These trials also include virotherapy, which was first studied specifically for anti-cancer purposes in 1991 [9]. Viral based therapies have been an ongoing area of interest in modern medicine [10,11]. A case of an acute leukemia patient who had temporary remission following an influenza infection was published in 1896 [10,11]. The advent of cell culture technologies in the mid-20th century led to the observations that viruses could infect and break down cancer cells, forming the foundation of our current understanding of oncolytic viruses [12,13,14]. Oncolytic viruses principally work through two mechanisms of action. Some oncolytic viruses infect and selectively replicate within tumor cells [15]. An alternative approach involves gene therapy, where viruses otherwise rendered replication-incompetent are administered to deliver transgenes which can promote an antitumor effect. Many studies have combined approaches, retaining the ability to replicate and lyse target cells while expressing a novel transgene [16,17,18,19,20]. Regardless of the approach, the overall goal is to generate direct cytotoxicity through viral replication or transgene expression. Depending on specific mechanisms for each antitumoral viral product, this may be followed by the administration of an activating drug or prodrug [16,17,18,19,20]. This cytotoxicity is then expected to generate an antitumor immune response, leading to improved tumor clearance and survival.

**Figure 1:**
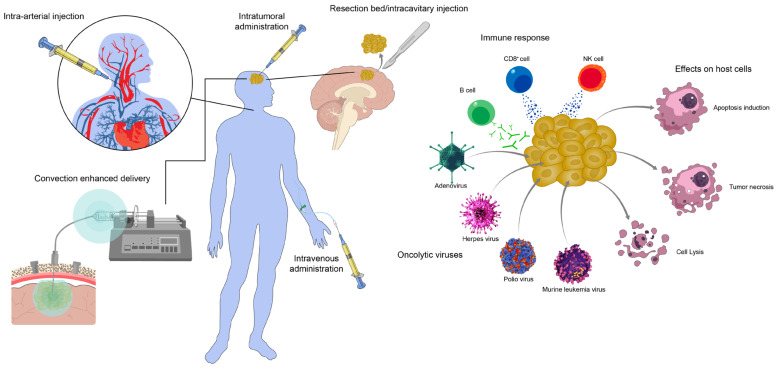
Overview of discussed delivery methods, specific oncolytic viral agents, and anti-tumor response for oncolytic viruses used in GBM therapy.

Virotherapy has been a subject of interest in GBM with various oncolytic and transgene approaches studied in multiple clinical trials. Here we discuss the current state of the field in virotherapy targeting GBM with an emphasis on promising viral therapeutics (Figure 1) or notable challenges to otherwise promising options.

## 2. Adenovirus-Based Therapies

### 2.1. Aglatimagene Besadenovec

*Mechanism*: Aglatimagene besadenovec (AdV-tk) is a non-replicating adenoviral vector containing the herpes simplex virus (HSV) thymidine-kinase (TK) gene [21]. AdV-tk is delivered via local injection, leading to HSV-tk expression by target cells. Anti-herpetic drugs, such as valacyclovir (VCV), acyclovir, and ganciclovir (GCV), are subsequently administered. Prodrugs are ultimately converted to acyclovir and mono-phosphorylated at HSV-tk expressing cells, generating a toxic nucleotide analog [22,23]. Using the same principle of conventional DNA-damaging chemotherapy, increased susceptibility is seen in replicating cells, such as the targeted tumor cells, with reduced effect in quiescent cells. As AdV-tk is injected locally, HSV-tk expression is expected to be predominantly limited to the tumor area and its immediate environment.

*Translational studies*: AdV-tk has been examined in a wide variety of malignancies. Safety has been broadly established in six phase I trials in patients with recurrent glioma [24], metastatic liver cancer [25], mesothelioma [26], retinoblastoma [27], recurrence ovarian cancer [28], and pancreatic cancer [29]. The first human trials of AdV-tk in patients with glioblastoma (GBM) were carried out between 1996 and 1998 [24]. Thirteen patients with recurrent glioma were enrolled, of which nine had GBM (Table 1). Patients were treated via single intratumoral injection of three logarithmically escalating doses of AdV-tk followed by 5 mg/kg GCV given intravenously 24 h after vector injection, then every 12 h thereafter for a goal of 28 doses. Toxicity was reached at a dose of 2 × 10^12^ viral particles (VP), with both patients treated at this dose demonstrating fever, encephalopathy, and hyponatremia. Further dosing was reduced to 2 × 10^11^ VP. Median post therapy survival for GBM patients was 4.0 months (16.8 months OS after initial diagnosis), with a range of 1.1–29.2 months (10.1–47.6 months after initial diagnosis).

A phase Ib study (NCT00751270) was next conducted for patients with malignant gliomas utilizing AdV-tk followed by VCV [32]. Twelve patients with presumed malignant glioma enrolled and completed experimental therapy, of which 10 had GBM and 2 had anaplastic astrocytoma (AA). AdV-tk was administered at the time of surgery into the residual tumor or resection bed in a total of 10 injection sites at least 1 cm apart, with 2 gm VCV started TID 1–3 days after surgery for a total of 14 days and standard of care.

(SOC) RT started 3–7 days after surgery and SOC TMZ after VCV was completed. Three dose levels were used (Table 1) and DLT was not observed. Adverse events thought to be related to AdV-tk were fever, wound complications, transaminitis, increased serum creatinine, confusion, cranial neuropathy, speech impairment, and headache. Median post therapy survival for all GBM patients was 10.9 months with a range of 2.0–46.4 months.

The phase IIa study NCT00589875 was next for patients with malignant glioma utilizing AdV-tk followed by VCV [30]. The 12 patients reported as part of NCT00751270 were also included in this study. Thirty-six additional evaluable patients were enrolled in this study, of which 34 had GBM and 1 had AA and 1 had anaplastic oligodendroglioma (AO). Methods were the same as NCT00751270, except IV acyclovir (10 mg/kg) was substituted in patients unable to receive PO medication and 3 × 10^11^ vp of Adv-tk was given to all patients. No DLTs were observed, with the most common AEs possibly attributable to AdV-tk including fatigue, fever, and headache. Median post therapy survival for all GB patients was 16.7 months.

NCT00634231 was a phase I trial in pediatric patients (ages 7–17) with malignant glioma, six of which had GBM [31]. AdV-tk and VCV administration were conducted as in NCT00751270 and NCT00589875, with the exception that TMZ was optional and additional investigator choice therapies could be included 8 weeks after AdV-tk administration. There were two dose levels, 1 × 10^11^ and 3 × 10^11^ vp, and no DLTs were observed. The most common AEs possibly attributable to AdV-tk included fatigue, fever, and nausea/vomiting. Median post therapy survival for all GB patients was 15.9 months, with a range of 7.4–37.3 months. The higher dose level of 3 × 10^11^ vp was associated with a higher median OS of 25.3 months compared to 8.9 at the low dose; however, this comes with the caveat that only three patients were treated at DL 1 and 5 at DL 2.

The phase II study NCT00870181 was conducted for adult recurrent high-grade glioma (HGG) patients utilizing AdV-tk followed by GCV [33]. A total of 22 patients received AdV-TK, 14 of which had recurrent GBM, and the remainder had AA or AO. Patients were treated with an intra-arterial cerebral infusion of AdV-TK followed by systemic GCV 5 mg/kg every 12 hours for 14 days. Mannitol was given prior to AdV-tk and GCV administration in order to disrupt the blood-brain barrier and AdV-tk/GCV treatment cycles were repeated every 21 days for at least two total cycles. The most common AEs possibly related to AdV-tk treatment included fever and headache. One patient had cerebral vasospasms and several developed neutropenia (grade 1–3). The median OS for GB patients was 10.4 months with a range of 2.1–54.9 months.

Currently active since 2018 is the phase I clinical trial NCT03576612 utilizing AdV-tk + VCV. Patient selection focuses on those with malignant glioma with treatment to be administered to the resection bed or residual tumor. A maximum of 36 patients have been allotted to this study.

### 2.2. Adenoviral RheoSwitch Therapeutic System Human Interleukin 12

*Mechanism*: Although cytokine-based therapies were initially thought to be promising options for novel cancer therapeutics, early studies showed intolerable side effects from systemic administration [50]. One such cytokine, interleukin 12 (IL-12), had been shown to enhance effector immune cells, including CD8 T cells [51], but clinical trials were similarly thwarted by toxicity [52]. In an effort to overcome this challenge, a gene delivery platform technology, RheoSwitch Therapeutic System (RTS), was developed using a replication-incompetent adenoviral vector with an IL-12 transgene [53]. Transgene expression is regulated by veledimex (3,5-dimethyl-benzoic acid [R]-N-[1-tert-butyl-butyl]-N′-[2-ethyl-3-methoxy-benzoyl]-hydrazide) (VDX), which can be given orally. The treatment hypothesis for the platform was that the adenoviral RTS with an interleukin 12 transgene (Ad-RTS-IL-12) could be injected into the target tumor, leading to uptake and transgene expression, mostly limited to the target neoplasm. Without VDX, fusion proteins are constitutively expressed and generate an ‘off’ signal without transgene expression [20]. When given, VDX, which was shown to have approximately 50% efficacy in its ability to cross the blood brain barrier [34], stabilizes a heterodimeric complex between the two fusion proteins and leads to transcriptional activation. Since the initial Ad-RTS-IL-12 vector is injected locally and cannot replicate, transgene expression of IL-12 following VDX is thereby limited to the proximity of the initial injection. This methodology was validated in GL-261 orthotopic murine models of glioma and non-human primate models before moving to human trials [20,53].

*Translational studies*: The first human clinical trial with adenoviral RTS with human interleukin 12 transgene (Ad-RTS-hIL-12) was posted in 2011 for patients with melanoma (NCT01397708), with later trials in breast cancer (NCT01703754, NCT02423902). The first clinical trial in glioblastoma patients was posted in 2014 (NCT02026271) [34]. This was a phase I trial in patients with recurrent or progressive GBM/malignant glioma. A total of 31 patients were treated, 28 of whom had a diagnosis of GBM. Ad-RTS-hIL-12 was freehand injected into two peritumoral sites of the post-surgical bed following tumor resection at a dose of 2 × 10^11^ vp. Dose escalation was conducted with VDX, which was given at 10, 20, 30, and 40 mg. A single dose was given 3 h before resection, then restarted on postoperative day 1 and given daily for 14 days. Correlative studies were conducted, demonstrating proof of concept, which showed VDX in the resected tumor tissue and elevations in both serum IL-12 and IFN-γ in patients taking VDX. The 30 and 40 mg dose levels of VDX were poorly tolerated, demonstrating high rates of cytokine release syndrome (CRS), transaminitis, and lymphopenia. Most patients tolerated the 20 mg dose, though CRS remained a common side effect. The median OS for all patients receiving 20 mg VDX (15 patients) was 12.7 months. The longest surviving patient was alive at 30 months with the low bounding survival range not given. Isocitrate dehydrogenase (IDH) status was also evaluated, with 20 patients having IDH-wildtype (wt) GBM, 5 IDH-mutant (mut) GBM, and 3 not specified. Despite this, the authors report no significant difference in OS when evaluating IDH status.

NCT03636477 was a phase I trial of Ad-RTS-hIL-12 and VDX in combination with the anti-PD-1 antibody nivolumab in patients with recurrent GBM [35]. A total of 21 GBM patients were treated. Nivolumab was given IV to patients 7 days before surgical resection, with Ad-RTS-hIL-12 and VDX given as in NCT02026271. Nivolumab was resumed at day 15 post-resection and then every two weeks thereafter. There were three dosing cohorts, with Ad-RTS-hIL-12 always given at 2 × 10^11^ vp: (1) Ad-RTS-hIL-12 + VDX 10 mg + nivolumab 1 mg/kg (*n* = 3), (2) Ad-RTS-hIL-12 + VDX 10 mg + nivolumab 3 mg/kg (*n* = 3), (3) Ad-RTS-hIL-12 + VDX 20 mg + nivolumab 3 mg/kg (*n* = 15). Pharmacokinetic analysis of intratumoral VDX and peripheral concentrations of IL-12/IFN-γ were also conducted, re-affirming prior results [34]. AEs were common, with nine patients experiencing grade 3 or higher toxicities, the most common of which were decreased lymphocyte count, brain edema, transaminitis, and CRS. However, most AEs resolved by withholding either VDX or nivolumab. Median OS for all patients was 9.8 months with a range of approximately 1–24 months. IDH status was evaluated with 19 patients having IDH-wt GBM and 2 IDH-mut.

Two additional studies with Ad-RTS-hIL-12 and VDX have completed recruitment with the publication of final results pending. The first, NCT03679754, is a phase I trial of recurrent or progressive GBM. An abstract from ASCO 2020 has been published [36] with preliminary information. Ad-RTS-hIL-12 and 20 mg of VDX were given as previously described, with 36 GBM patients treated. Median OS for a sub-population of 20 patients with unifocal disease and less than 20 mg of dexamethasone given while on VDX was 16.2 months. The second, NCT04006119, is a phase II trial of recurrent or progressive GBM. Distinct from prior trials is the addition of cemiplimab-rwlc (Libtayo), a PD-1 antibody. An abstract from SNO 2021 has been published with preliminary information [37]. A maximum of 40 patients have been allotted to this study and the treatment schedule is the same as NCT03636477, with the exception that cemiplimab-rwlc (350 mg IV) takes the place of nivolumab and is given every three weeks rather than two. 

### 2.3. Tasadenoturev

*Mechanism*: DNX-2401 (Delta-24-RGD; tasadenoturev) is a conditionally replicative adenovirus that uses a 24-base pair deletion in the E1 region. E1a proteins are required for viral replication, as they are the first polypeptides synthesized following adenoviral infection [54,55] E1a proteins target specific proteins, amongst which is the retinoblastoma gene (Rb). By deleting the 24 bp region, the formation of E1A/Rb complexes can be prevented. Therefore, this modified virus will not efficiently infect normal cells, which are competent in their Rb pathway, but rather will infect and replicate in Rb or Rb regulatory protein deficient cells [56]. DNX-2401 also has been engineered with an RGD-motif in the fiber H-loop. This enhances tumor cell selectivity by enabling DNX-2401 to use αvβ3 or αvβ5 integrins, commonly present on glioma cells [57,58]. Preclinical studies were largely conducted in murine models, including intracranial frontal lobe xenografts in nude mice [58,59].

*Translational studies*: Given the RGD-motif, DNX-2401 has largely been explored in gliomas. There has been some promise in non-GBM neoplasms, specifically pediatric diffuse intrinsic pontine glioma [60]. The first trial encompassing GBM was posted in 2008 (NCT00805376) [38]. This was a phase I trial in 37 (33 GBM) patients with recurrent malignant glioma. Patients were further subdivided into two arms, the first of which was 25 patients who received recurrence-confirming biopsy, then single IT injection of escalating doses of DNX-2401. The second arm of 12 patients was treated as the first arm, but with an implanted catheter used for the injection of DNX-2401 and the tumor was resected 14 days later, followed by additional DNX-2401 injection in the resection bed. Dose escalation used a 3 + 3 design starting at 1 × 10^7^ vp to a maximum of 3 × 10^10^ vp. No DLTs were observed and 3 × 10^10^ vp, the highest dose given, was deemed the MTD. Adenoviral DNA was detected in less than 3% of collected patient samples from serum, sputum, or urine and AEs attributed to DNX-2401 were grade 1 to 2 headache, nausea, confusion, vomiting, and fever. Tumor volume reductions were seen in 72% of patients in arm 1, with five patients having greater than 3-year OS. The median OS for all GBM patients was 9.8 months with a range of 2.3–57.9 months. The IDH status demonstrated that 10 patients had IDH-wt GBM and 2 IDH-mut GBM, with the remaining GBM patients not evaluated.

Two clinical trials for DNX-2401 in GBM patients were posted in 2012 and 2013, respectively (NCT01582516, NCT01956734). The former is a phase I/II trial in up to 20 patients with recurrent GBM with DNX-2401 to be administered by convection enhanced delivery (CED). The latter is a phase I in up to 31 patients with first recurrent GBM with DNX-2401 to be administered intratumorally or into the resection bed and TMZ starting every 14 days following viral administration. Both trials have completed recruitment with the publication of results pending. Another phase I clinical trial, called TARGET-I, was posted in 2014 (NCT02197169), enrolling up to 37 patients with recurrent GBM or gliosarcoma with intratumoral DNX-2401 and systemic IFN-γ. Preliminary results from this study were presented at the American Society of Clinical Oncology (ASCO) 2017 meeting [39]. The abstract notes that 27 patients were treated, 18 of whom received DNX-2401 followed by IFN-γ and 9 with DNX-2401 alone. DNX-2401 was given at 3 × 10^10^ vp, while IFN-γ was given subcutaneously at 50 mcg/m^2^ starting at day 14 and then every three weeks thereafter. They noted that IFN-γ was poorly tolerated and had no survival benefit over DNX-2401 alone. The most common AEs grade 3 or higher were fatigue, headache, and seizures. Twelve- and 18-month OS rates were 33% and 22%, regardless of the treatment arm. The median OS was not reported. In a subsequent phase 2 study for patients with recurrent GBM or gliosarcoma (NCT02798406), intratumoral DNX-2401 administration is followed by IV pembrolizumab, starting 7–9 days post-operatively and continuing every 3 weeks. Results from this trial are pending.

Results from a phase I study based in the Netherlands using DNX-2401 were published in 2022 [40]. A total of 20 patients with recurrent GBM were enrolled, 19 of whom received DNX-2401. Patients who did not undergo resection underwent convection enhanced delivery (CED) through four catheters, two within the tumor and two peritumorally. Patients who received resection did so 1 week prior to CED infusion. Infusions were completed over a time period of 44 to 67 h. Dosing started at 1 × 10^7^ vp with escalation planned to 1 × 10^11^ vp. The maximum administered dose was 3 × 10^10^ vp with no further escalation recommended due to a CSF leak in a patient at that dose. Treatment-related AEs included confusion, seizure, increased intracranial pressure, neurological deterioration, meningitis with hydrocephalus, and wound dehiscence. The median OS reported was 4.2 months with a range of 2.2–91.8 months. One patient had a complete response without recurrence or additional treatments. The IDH status demonstrated that 15 patients had IDH-wt GBM and 1 IDH-mut GBM, with the remaining GBM patients not evaluated [40]. The patient that had this complete response with long-term survival had an IDH-mut GBM and received the highest dose at 3 × 10^10^ vp.

There is additionally an active and recruiting phase I clinical trial, NCT03896568, with DNX-2401 for patients with recurrent HGG. A maximum of 36 patients have been allotted. Unique to this trial, allogeneic mesenchymal stem cells (MSC) are planned to be loaded with DNX-2401 prior to intra-arterial injection. Results are not yet available.

### 2.4. NSC-CRAd-S-pk7

*Mechanism*: CRAd-Survivin-pk7 is a conditionally replicative adenovirus (CRAd) with a human survivin promoter (S) which drives E1 expression and a polylysine sequence modification of the fiber knob (pk7), allowing selective binding to heparan sulfate proteoglycans (HSPGs) [61]. This modified virus was developed in response to challenges in adenoviral transduction into human tumors, which limited the efficacy of adenovirus as an oncolytic [62]. However, methods to increase adenoviral effectiveness can potentially lead to unacceptable off-target toxicity. One way to circumvent this issue is to specifically target replication and oncolysis to neoplastic tissue by incorporating a tumor-specific promoter within the E1 region, which is critical for viral replication, as previously described [54,55]. Survivin is a member of the inhibitor of apoptosis protein family [63]. It is typically present during embryogenesis and otherwise undetectable in normal tissue. By incorporating survivin as a promoter within the E1A region of the virus, replication is targeted to the element (survivin) which is over-expressed in malignant tissue, including gliomas, and thereby leveraged as an oncolytic [63]. Additionally, the CRAd-Survivin-pk7 was modified at its fiber knob to enhance HSPG binding. These HSPGs are overexpressed in glioma [64], thereby improving adenoviral tropism for glioma tissue [65]. Overall, this modified virus should therefore have selective oncolytic and replicative potential in survivin and HSPG expressing cells, both of which are common in malignant glioma.

Neural stem cells (NSCs) were examined as a mechanism to deliver the virus, given their capacity to bypass the BBB [41]. Further, there is evidence that NSCs demonstrate some selective tropism for CNS malignancy, making it a promising mechanism for therapeutic delivery. The FDA approved the NSC cell line; HB1.F3.CD21 was studied in murine models, demonstrating the capability of NSCs to deliver the viral load to glioma tissue [66]. With promising preclinical findings, human clinical trials were pursued.

*Translational studies*: NCT03072134 was the first clinical trial of NSC-CRAd-S-pk7 [41]. In this phase I trial, the oncolytic was studied in combination with standard radiation and chemotherapy for patients with newly diagnosed malignant glioma. A total of 12 patients were enrolled, 11 of which had GBM. The initial diagnosis was confirmed at the time of surgery and NSC-CRAd-S-pk7 was injected into the resection bed at up to 10 sites. Dosing was conducted in a 3 + 3 design with three total dose levels. DLT was not observed, and the maximum dose was recommended for future clinical trials. AEs attributed to NSC-CRAd-S-pk7 administration were meningitis, cerebral edema, encephalopathy, and subdural fluid collection. The median OS for all patients was 18.4 months. IDH status demonstrated that 10 patients had IDH-wt GBM and 2 IDH-mut GBM.

NCT05139056 is an active and recruiting phase I clinical trial for patients with recurrent HGG using NSC-CRAd-S-pk7. A maximum of 36 patients have been allotted. The listed delivery mechanism is intracavitary infusion following surgical resection and, thereafter, weekly treatment for up to four doses. Study results are pending publication.

## 3. Herpes Simplex Virus-Based Therapies

### 3.1. M032-HSV-1

*Mechanism*: M032 is a second-generation oncolytic herpes virus (HSV) [67]. HSV viruses hold particular promise for the treatment of glioma given the proclivity of HSV for neural tissue. Replication for M032 is prevented in nonmalignant cells by the deletion of the neurovirulence gene *γ_1_34.5* [68]. Over time, multiple iterations have been examined, including G207, HSV1716, and M002, with the goal of further enhancing safety or efficacy. M002 was designed with the *γ_1_34.5* deletion and murine p35 and p40 subunits of IL-12 in order to promote IL-12 expression in treated tumor tissue [69,70], which showed pre-clinical efficacy in murine models. M032 is identical to M002 with the exception that it expresses human IL-12 p35 and p40 subunits. This was shown to be safe in nonhuman primate models before moving to human clinical trials [67].

*Translational studies*: Initial human trials started in 2014 (NCT02062827) [18]. This is a phase I clinical trial in patients, up to a maximum of 24, with recurrent GBM, AA, or gliosarcoma. This trial remains active but is no longer recruiting. M032 is infused into a catheter into the tumor at doses of 1 × 10^5^, escalating by logs as tolerated to a maximum of 1 × 10^9^. AEs are expected to be consistent with those seen in M032’s cousin virus, G207 [71,72,73]. Additionally, there is also currently an active and recruiting phase I/II clinical trial of M032 in combination with the PD-1 antibody pembrolizumab for patients with either newly diagnosed or recurrent GBM, AA, or gliosarcoma. The dose of M032 to be utilized in this study will be the MTD determined from the phase I study NCT02062827. A maximum of 28 participants have been allotted, with IV pembrolizumab to be started at week 4 and every three weeks thereafter.

### 3.2. HSV-1 Virus rQNestin34.5v.2

*Mechanism*: rQNestin34.5v.2 is another oncolytic HSV (oHSV) being explored as a treatment for glioma [74]. This oHSV also exploits the HSV1 neurovirulence factor *γ_1_34.5* [68]. However, rather than creating a deletion which eliminates functionality, rQNestin34.5v.2 restores one copy of *γ_1_34.5* with transcriptional control under a nestin promoter [75,76]. The hypothesis behind this is that with the elimination of *γ_1_34.5*, viral evasion, and replication capacity is significantly hampered. As nestin is overexpressed in a variety of neoplasms, including glioma, this neurovirulence gene expression should be limited to nestin expressing neoplasms. This version, rQNestin34.5, was modified further with deletion of a fusion transcript made by sequences encoding for green fluorescent protein (GFP), creating rQNestin34.5v.2. Initial studies in mice showed reduced toxicity when compared to a wild-type viral strain, after which human trials were proposed [74].

*Translational studies*: Currently, rQNestin34.5v.2 is in an active and recruiting phase I clinical trial (NCT03152318). A 2021 ASCO abstract was published which describes initial results [42]. Eligible patients were those with multifocal, multicentric, tumors larger than 5 cm or recurrent tumors. A maximum of 62 patients were allotted, with 30 patients (26 GBM) treated at the time of abstract publication. rQNestin34.5v.2 was injected IT at 1 × 10^6^ pfu, with future patients escalating in a 3 + 3 model with dose escalation by half-logs to a maximum of 1 × 10^10^ pfu. Patients were also eligible for standard of care treatments. The median OS for all 30 patients was 13.25 months. There is an additional arm listed in the trial for patients to additionally receive IV cyclophosphamide (CTX) 2 days prior to surgery. 

### 3.3. Teserpaturev

*Mechanism*: Teserpaturev (G47Δ) is a conditionally replicative oncolytic herpes simplex virus type 1 [77]. G47Δ is derived from G207. G207 has deletions in both copies of the neurovirulence *γ34.5* gene and an inactivating insertion in the *UL39* gene, which encodes the infected cell protein 6 (ICP6) [78]. In addition to the effects that deletion of the *γ34.5* gene have as previously described, the *UL39* gene mutation also generates preferential replication in dividing cells. ICP6 is a ribonucleotide reductase enzyme critical for nucleotide metabolism and viral DNA synthesis in nondividing cells, thereby severely attenuating viral efficacy for nondividing, non-neoplastic tissue [79]. What differentiates G47Δ from G207 is the additional deletion of the *α47* gene and the promoter region of *US11* [80]. This creates a two-fold benefit. First, *α47* is a mediator of viral escape, as it downregulates MHC class I expression in infected host cells, and deleting this gene led to retained MHC I expression and higher cytotoxicity both in vitro and in vivo. Secondly, the deletion of the *US11* promoter leads to the placement of the *US11* gene under the *α47* promoter. This generates partial recovery of functions of the deleted *γ34.5* gene, generating improved viral replication [81]. The result is an oncolytic virus with tropism for tumor cells demonstrating improved replication and the ability to generate a sustained immune response. This virus has been studied in human clinical trials including prostate adenocarcinoma [82], metastatic breast cancer [83], gastric cancer [84], tongue cancer [85], and esophageal cancer [86] with initial promising results.

*Translational studies*: The first clinical trial of G47Δ in GBM, UMIN000002661, was a phase I/II trial of 13 patients with recurrent GBM [43]. There were two dose levels and G47Δ was given intratumorally twice, the second dose at identical coordinates within 5–14 days of the first injection. A third dose level was not advanced, as the 1 × 10^9^ pfu dose had three patients which experienced convulsions. The most common AEs were headache, fever, and vomiting, with AEs thought possibly due to G47Δ including headache, fever, vomiting, convulsions, nausea, intratumoral hemorrhage, wound pain, anorexia, decreased leukocyte count, anemia, tremor, and cranial nerve disorder. Viral shedding from blood, urine, and saliva was consistently negative. The median OS was 30.5 months from initial diagnosis and 7.3 months with a range of 3.2 to 143.9 months from the last G47Δ administration. In post-hoc immunohistochemistry, two patients were noted to have an IDH mutation.

The next clinical trial, UMIN000015995, was a phase II study conducted in 19 patients with residual or recurrent GBM [44]. G47Δ was injected intratumorally at 1 × 10^9^ pfu for up to a total of six doses at 5–14 days for the first two doses and 4 +/− 2 weeks for subsequent doses. All patients (100%) had G47∆-related adverse events, the most common G47∆-related AEs being fever, vomiting, nausea, decreased lymphocyte count, and decreased white blood cell count. Fever was the only serious AE, with one patient requiring prolonged hospitalization. Viral shedding from blood, urine, and saliva samples was negative at all time points with the exception of a single patient on day 0 only. The median OS after initial surgical diagnosis was 28.8 months and 20.2 months with a range of 4.2–65.3 months after G47Δ initiation. In post-hoc immunohistochemistry, six patients were noted to have an IDH mutation, however OS was not affected by IDH status.

## 4. Polio Virus-Based Therapies

### Recombinant Nonpathogenic Polio-Rhinovirus Chimera

*Mechanism*: Recombinant nonpathogenic polio-rhinovirus chimera I (PVSRIPO) is a live attenuated poliovirus type 1 (Sabin) vaccine [45]. PVSRIPO is a hybrid virus, with its internal ribosomal entry site replaced with that of a human rhinovirus type 2 order to diminish neurovirulence [87]. Despite this decreased neurovirulence, this hybrid virus still maintained propagation in glioma cell lines [88]. This is in part due to human poliovirus receptor, CD155, which is commonly expressed on malignant gliomas [89]. In addition to oncolysis, PVSRIPO also activates dendritic cells and produces a cytotoxic, IFN-γ driven response with initial efficacy in an immunocompetent murine melanoma model [90]. 

*Translational studies*: NCT01491893 was a phase I clinical trial studying PVSRIPO first posted in 2011. A total of 61 patients with recurrent GBM were treated [45]. Following stereotactic biopsy, PVSRIPO was infused into the tumor via CED over 6.5 h with no additional resection thereafter. A total of seven dose levels were given, with dose reduction to dose level (DL) −1 and −2 in the dose expansion phase (5 × 10^7^ and 1 × 10^7^ TCIDs). DL −1 was identified as the phase 2 dose. AEs potentially attributable to PVSRIPO included vision changes, nausea, vomiting, fatigue, gait disturbance, seizure, per minimal trach syndrome, paresthesia, headache, dysphagia, and confusion. One DLT occurred at the highest dose level with intracranial hemorrhage following catheter removal necessitating surgical evacuation and complicated by right hemiparesis and aphasia. In the dose expansion phase of 52 patients at DL −1, 19% had grade 3 or higher AEs. The median OS for all 61 patients was 12.5 months with a range of 3.1–70.4 months. In total, 21% of patients survived to 36 months. The IDH status demonstrated that 45 patients had IDH-wt GBM and 7 IDH-mut GBM, with 9 patients having an unknown IDH status. Survival analysis conducted by the authors to evaluate for possible survival advantage of IDH-mut patients showed no additional survival benefit based on the IDH status.

Following these results, there have been a number of posted clinical trials that are active but not currently recruiting. These include NCT02986178, a phase II trial for recurrent malignant glioma, NCT03043391, a phase Ib trial for pediatric patients with recurrent malignant glioma, and NCT04479241, a phase II trial with the addition of pembrolizumab to PVSRIPO for patients with recurrent GBM. All propose to utilize CED. An expanded access trial using PVSRIPO posted in 2020 (NCT04599647) for patients with GBM is no longer available.

## 5. Murine Leukemia Virus-Based Therapies

### Vocimagene Amiretrorepvec

*Mechanism*: Vocimagene amiretrorepvec (Toca 511) is a non-lytic, replication-competent murine leukemia virus [91]. It is a retrovirus that selectively targets dividing cells and has highly efficient gene delivery in preclinical models, with initial studies showing that multiplicities of infection as low as 0.0001 were able to replicate throughout an entire tumor model [92]. The virus was engineered to encode a yeast cytosine deaminase (CD), such that any cell infected with Toca 511 would also express CD. In cells expressing CD, the pro-drug 5-fluorocytosine (5-FC) would be converted to the drug 5-fluorouracil (5-FU), a potent chemotherapeutic agent [93]. 5-FU is an antimetabolite and thereby has a predominant effect on dividing cells, the basis for its continued use as a cancer therapeutic. Initial studies in murine glioma models demonstrated that by treating with Toca 511 and 5-FC, it was possible to stably express the pro-drug activating gene within the tumor and generate long-term survival benefits [91].

*Translational studies*: The first human study for Toca 511 was a phase I posted in 2010 (NCT01156584). Preliminary data from 36 treated patients were published in an abstract in 2014 [46]. Toca FC (5-FC) was given once a month after completion of Toca 511 therapy. No DLTs were observed. Phase I clinical trial, NCT01470794, was posted in 2011 [47]. Toca 511 was injected at escalating doses into the resection bed of patients with FC given orally at 6 weeks after surgery. A total of 56 patients were treated, 46 had GBM. Specific doses for Toca 511 and Toca FC were not published. A total of two patients had AEs leading to treatment discontinuation. Median survival for all evaluable patients (n = 53) was 11.9 months. While full patient details on IDH status were not published, authors did note that of the patients with complete responses, two were IDH-mut and three IDH-wt. In another phase I trial, NCT01985256, Toca 511 was given IV prior to recurrent HGG resection in addition to resection bed injection [48]. Ten patients were treated at the time of abstract publication. No AEs or DLTs have been reported.

NCT02414165 was a phase II/III clinical trial for patients with recurrent GBM or AA which was ultimately terminated for futility. A total of 403 patients were randomized to standard of care or Toca 511/FC, of which 201 were in the treatment arm and 171 had GBM [49]. Toca 511 was injected into the resection cavity at a dose of 4 × 10^8^ TU, with Toca FC given at 220 mg/kg/day. The median OS was 11.1 months for the Toca 511/FC arm and 12.2 months for the standard of care arm. The IDH status demonstrated that 345 patients had IDH-wt GBM and 58 IDH variants. Toca 511/FC did not improve OS in recurrent GBM or AA patients with the study terminated for futility. It was later noted that median prodrug dosing was suboptimal in the phase II/III study [94]. Two additional studies investigating Toca 511, the Ib NCT02598011 and II/III NCT04105374, were subsequently withdrawn.

## 6. Discussion

To date, multiple viral oncolytic agents have been studied for the treatment of HGG in general and GBM in particular [20,24,32,38,41,44,45]. These viral oncolytics are designed for selective uptake or replication in tumor cells. The treatment mechanism then varies according to the specific engineered virus. Many work through direct, selective cell lysis, with the engineered virus designed to favor oncolysis over non-malignant cellular targets [15]. Others utilize antiviral prodrugs following viral administration with the goal of exerting a cytotoxic effect on infected tumor cells. Alternatively, replication-incompetent viruses have been administered for selective uptake by tumor cells to generate anticancer cDNA. This is then followed by the administration of a prodrug that is converted into an active anticancer drug within tumor cells, with the goal of having a direct tumor-specific cytotoxic effect [24,30]. The initial cytotoxic effect is expected to release antitumor antigens, resulting a sustained adaptive antitumor immune response. This review highlights the fact that there are multiple OV types, as well as multiple arming strategies which have shown sufficient pre-clinical promise to make it into clinical trials, but which have not translated such promise into human trials. We highlight these failures not as an indication that OVs are not promising, potentially revolutionary agents for the treatment of GBM and HGG. Rather, we are concerned that, as the early phase trial data accumulates, the lack of convincing clinical benefits may turn opinion against this entire class of therapeutic agents on the grounds that no single variation has proved effective. While the lack of conversion of exciting pre-clinical data into clinical success is by no means unique to the OV field—and the clinical trials summarized above are still largely in the early phase—we propose that reflection and innovation is required to revitalize OVs as a feasible contender for GBM therapeutics.

*Oncolytic virotherapy is no longer new*: It is not uncommon to read manuscripts which describe oncolytic virotherapy as a new and promising strategy for the treatment of cancer. This review helps to put those comments somewhat into perspective. The oldest trial we describe in this review, NCT00589875 with AdV-tk, started in 2008 [30]. It is clear from the abundance of clinical trials that OV results have now been around for long enough that perhaps they can no longer be considered emerging, ground-breaking therapies. This means that they should no longer be viewed as within a honeymoon phase, where results that are available, or still pending, are interpreted with the leeway that usually comes with such a period. It is clear from the above that clinical results are now available and should be subject to scrutiny. In this respect, this thorough review of clinical data of OV trials in patients with GBM is somewhat underwhelming in terms of standout efficacy. Despite a range of viruses being tested in patients, some of which are armed with additional cytokine genes, there are only a smattering of individual patients who seem to have done impressively well clinically. Obviously, many of these trials are still in the early phase and true efficacy awaits larger scale phase II/III results. Nonetheless, in the spirit of post-honeymoon reality, perhaps it is now time to reassess the role of OV monotherapy in GBM.

*As yet, there are no large-scale clinical trials showing efficacy of oncolytic virotherapy as a monotherapy*: Despite their conceptual promise, there are no successes from large scale clinical trials utilizing viral therapeutics. The largest study to date, which involved Toca 511 and Toca FC for patients with recurrent GBM and AA, was terminated for futility [49]. There was no improvement in overall survival among patients receiving the viral therapeutic compared to standard of care options such as bevacizumab, temozolomide, or lomustine. The remaining viral therapeutics have been predominantly studied in small phase I clinical trials, with tolerable toxicity profiles but no large successes leading to widespread adaptation. The failure of TOCA511 and the lack of any outstanding cohorts with several different OV in trials should make us take stock of how these viruses can best be used to the benefit of patients with glioma. With new viruses being tested pre-clinically, and multiple viruses with multiple arming strategies being used successfully in those pre-clinical studies, a reassessment of the criteria for ultimate efficacy may now be warranted. Why are these viruses not getting breakthrough results? Is it that the trials are not yet mature enough to see such results, or are there fundamental issues at play that we need to address? Are there really subsets of patients who will do well and how do we identify them? As our critical review of the trial data shows, it seems unlikely that all the viruses proposed for the treatment of GBM can be effective. The hope is that this does not represent an ‘Emperor’s New Clothes’ scenario—in which no one was prepared to own up to the fact that the emperor—who thought he was wearing beautiful clothes—was actually naked. The major question for the future is how to uncover the conditions under which any (or even possibly all) of the plethora of viruses currently being tested, or which will be tested in the future, can be made to work for the benefit of patients. 

That is not to say that there are no data suggesting that OV can impact patient care. For example, five patients treated with DNX-2401 had greater than 3-year OS [38]. The recent phase I and II trials of Teserpaturev demonstrate an impressive median survival benefit over historical controls [43,44]. However, do these patients represent real successes for whom the constellation of tumor type, location, and individual geno/phenotype matched the properties necessary for successful viral replication and immune priming of anti-tumor immunity? Or do they represent outliers who did well clinically because of the standard of care they received while on trial or other non-virus-related factors? Answers to this type of question will come from developments in trial design and especially in the execution of high powered, critical virological and immunological correlative assays so that the correlates of clinical efficacy can be accurately matched to patient and tumor geno- and phenotypes. In this respect, major advances in the understanding of how and when OV can be used to the best effect in patients are likely to come from the discovery of biomarkers associated with success. In this way, patient selection will improve with the resultant improvement in clinical results.

*Challenges to oncolytic virotherapy as a monotherapy*: Multiple challenges have been hypothesized as the etiology of disappointing results in OVs for gliomas. These include unoptimized viral delivery mechanisms, prodrug selection and adherence, and inadequate generation of sustained antitumor immunogenicity. In most studies to date, viral particles have been delivered by intratumoral injection following surgical resection. While this approach has the benefit of introducing viral particles directly into the tumor area, it is difficult to determine how much microscopic tumor is truly exposed to the injected viruses [94], particularly considering the presence of normal brain parenchyma or necrotic tissue representing a physical barrier. The degree of uptake by tumor cells is also uncertain, as is the propensity for the successful induction of replication. Approaches to improve viral tropism for tumor cells and the optimization of delivery methods by utilization of techniques such as convection-enhanced delivery may overcome these challenges [95].

Another challenge involves the dosing and timing of administered prodrugs. One concern with the Toca 511/Toca FC study involved the median number of prodrug cycles administered to patients. In preclinical studies, investigators demonstrated the need for 3–4 prodrug cycles to generate an anti-tumor immune response, whereas in the phase 3 clinical trial, patients received a median of only two cycles [94]. The improved optimization of timing and dosing of prodrugs in future studies may overcome this problem. The degree of initial cytotoxicity generated by the selected viral particle prodrug combination also requires careful consideration.

Intertumoral and intratumoral heterogeneity of GBM must be considered as this serves the foundation for therapeutic resistance [96,97]. Glioblastoma was the first cancer type systematically studied as part of The Cancer Genome Atlas Research Network (TCGA), gradually leading to the characterization of proneural, mesenchymal, and classical subtypes as well as proneural to mesenchymal transition described for recurrent tumors [98]. While greatly improving the understanding of GBM, subtype classification has yet to translate into effective treatment stratification or improved outcomes, in part due to intratumoral heterogeneity and phenotype switching [99,100,101]. The potential of viral therapeutics only treating subpopulations of tumor cells must be considered.

Emerging data also underscore the importance of the tumor microenvironment (TME) [102], which includes the extracellular matrix, microglia, tumor stem cells, mesenchymal stem cells, and tumor associated macrophages. Remodeling of the TME is possible and plays an important role in disease progression [103,104,105]. How the TME is altered by viral therapeutics as well as how the TME can be modulated by other interventions to optimize efficacy of viral therapeutics must be considered.

Finally, sustained response from viral therapeutics hinges on the generation of a durable antitumor immune response. The inherent immunosuppressive nature of GBM represents a great challenge in this regard. GBM and other HGGs are typically associated with a low mutational burden, with few antigens with which to elicit an antitumor immune response [7]. Paracrine immunosuppressive mediators released from GBM cells have been described [106]. Histological GBM specimens demonstrate few tumor-infiltrating lymphocytes with the existing cells additionally exhibiting an exhausted phenotype [107]. Additionally, GBM causes systemic immunosuppression via T cell sequestration [108]. For any viral therapeutic to generate a sustained antitumor immune response, these limitations must be overcome. Combinatorial approaches such the co-administration of viral therapeutics with immune checkpoint inhibitors or the use of cytokine-based therapies to increase peritumoral CD8 lymphocytes may help overcome these challenges [7,109].

*Oncolytic viruses are promising candidates for breaking tumor microenvironment immune suppression*: Strategies which turn immunologically barren, or ‘cold’, tumors into inflamed, or ‘hot’, tumors will make them amenable to direct immunological rejection, and/or to systemic immunotherapeutic interventions—such as combination with immune checkpoint blockade (ICB) or CAR T cell therapies. In this respect, oncolytic viruses are often quoted as the matches which could light the fire to bring immunological heat into cold tumors [110,111,112]. However, immunological heat comes in many different forms—ranging from ‘freezing cold’, through ‘tropical paradise’, and into ‘raging inferno’. Significantly, however, one immune cell type’s tropical paradise may represent another cell’s scorching inferno. Inflammation is a dynamic process involving initiation, resolution, and adaptation which links innate and adaptive immunity. There exists a myriad of immune players involved in the generation of an adaptive T cell response from an initiating infection and each one responds optimally to a very specific set of immune signals quantitatively, qualitatively, and temporally. Therefore, although viruses are ideal danger signals to alert the immune system, exactly how they can and should be used as immunological flame throwers needs to be very carefully determined.

Rapidly induced innate immune responses to pathogens, such as many types of OVs, are perfectly suited to the generation of local innate immune heat which can lead to the rejection of both infected and uninfected tumor cells [113,114]. If the therapeutic goal is simply to torch the tumor directly, with little thought of setting up a systemic anti-tumor T cell response, then many types of fully replicating, highly immunogenic OVs are very well suited. However, this type of raging innate immune heat is also highly likely to quench OV replication, working at odds to the goal of selectively replicative OV therapy [115]. Rapidly induced innate heat which responds to pathogens, such as OVs, is also ideal for the priming, activation, and propagation of adaptive immune responses to that pathogen. This occurs through a carefully regulated sequence of inflammation induction and subsequent active resolution, followed by a shift of chemokine and cytokine secretion towards a T cell tropic profile. However, innate, pathogen-associated, heat should not allow for the generation of adaptive immune responses to non-pathogen associated antigens, such as self-antigens, which may form the targets of autoimmunity. In this respect, OVs are often proposed to be able to prime—or boost—T cell responses against immuno-subdominant, weak, self-tumor antigens, or, at best, somewhat more immuno-dominant neo-antigens. The generation of these weak anti-tumor T cell responses must, however, occur in the presence of a developing T cell response against the virus itself, which often encodes multiple highly immunodominant viral epitopes. Thus, where the goal of the oncolysis is, at least in part, to prime weak anti-tumor T cell responses, it could be argued that antigen invisible, poorly immunogenic viruses should be selected to avoid the anti-tumor immune responses from being engulfed by very potent anti-viral responses. Once again, answers to these questions will come from well-designed trials which measure the ability of OV treatments to induce anti-viral and anti-tumor T and B cell responses, what the balance between the two is, and how this ties in with innate reactivities induced by the virus within the tumor microenvironment of GBM. 

*OV are excellent candidates for combination immunotherapy*: Oncolytic viruses are often quoted as being ideal adjuvant partners for combination with additional immune therapies, such as ICB or CAR T cell therapies [116,117]. This derives from their proposed activities to bring heat to tumors to attract adoptively transferred T cells, and from their ability to prime, or boost, anti-tumor T cell responses upon which ICB can act. However, whilst the innate immune heat induced by OVs is highly appropriate in the initiation of an adaptive immune response to the virus, and possibly against an infected tumor, innate immune responses to viral infection can be highly deleterious to pre-existing effector/memory T cells, including adoptively transferred CAR T cells [118]. Thus, innate immune heat, characterized by type I interferons and other cytokines, whilst a paradise for neutrophils and macrophages, is highly toxic to pre-activated effector T and CAR T cells. In addition, a successful combination of OV with ICB is predicated upon the ability of the OV either to boost a pre-existing anti-tumor T cell response in the patient, or to prime a de novo tumor antigen specific T cell response—upon which the ICB can then work to enhance the immune rejection of tumors. However, the use of OVs to induce genuine anti-tumor immunity, in the midst of a raging forest fire of anti-viral immunity, will require a very thoughtful application of viral induced heat, coupled with careful scheduling of the co-administration of ICB. In summary, the concept of turning cold tumors hot with OVs is extremely attractive to develop these agents as immunotherapies. However, not all immune heat is equal. We believe that a highly underappreciated aspect of the use of OVs as immunotherapies is that virus type, oncolytic goals, and immunological outcomes must be carefully mixed and matched.

## 7. Conclusions

Oncolytic viruses are now in clinical testing for the treatment of glioma. A variety of viruses have made it through the regulatory hurdles and into patients. Tantalizing clinical responses have been reported from individual patients, but no major signals have yet been seen. It is not clear if this is because of the predominantly early phase nature of the trials or if it reflects a more significant indication as to if, and how, these viruses might be valuable in this disease. The accumulation of trial data suggests that the honeymoon period for OV is coming to an end. A rational and realistic assessment of the field is now needed to address how so many viruses, with so many arming strategies, can all show such promise in the laboratory with, as of yet, no standout clinical signals of success. The path forward seems to us to hinge on identifying which patients/tumors will be susceptible to virus replication and immune priming; which viruses are mechanistically likely to be most suited to treat GBM; and how OV can best be used in rational temporal combination with other modalities. What does seem clear is that OV can no longer be thought of as the new kids on the block; there is real competition for success in the immuno-oncology space for HGG. While both CAR T cells and ICB are themselves still struggling to make an impact against this disease, for OVs to remain worthy candidates for success we must look at the emperor with realistic and critical eyes and be prepared to honestly say what we see.

## Figures and Tables

**Table 1:** OS: Overall survival; AdV-tk: aglatimagene besadenovec; GCV: ganciclovir; GBM: glioblastoma; AA: anaplastic astrocytoma; IT: intratumoral; inj: injection; vp: viral particles; VCV: valacyclovir; AO: anaplastic oligodendroglioma; Ad-RTS-hIL-12: adenoviral RTS with an interleukin 12 transgene; VDX: veledimex; CRS: cytokine release syndrome; dex: dexamethasone; cemiplimab-rwlc: Libtayo; DNX-2401: tasadenoturev; CED: convection enhanced delivery; TMZ: temozolomide; IFN-γ: interferon gamma; 12 m/18 m: 12 months, 18 months; ICP: intracranial pressure; MSC: mesenchymal stem cells; NSC: neural stem cells; CRAd-Survivin-pk7: conditionally replicative adenovirus with a human survivin promoter and a polylysine sequence modification of the fiber knob; HGG: high-grade glioma; pfu: plaque forming units; G47∆: teserpaturev; CN: cranial nerve; PVSRIPO: recombinant nonpathogenic polio-rhinovirus chimera I; TCID_50_: 50% tissue culture infectious dose; Toca 511: vocimagene amiretrorepvec; Toca FC: pro-drug, 5-FC; TU: transduction units; IV: intravenous; * OS for GBM patients only; ^§^ combined from NCT00751270 for total of 48 patients; ^ǂ^ OS survival after trial intervention. N/A: not applicable.

Virus	Clinical Trial Number	Phase	Dates	Status	Patient Population	Delivery	Dosing	Median OS (Months)	Range OS (Months)	Adverse Events	Citation
AdV-tk + GCV	N/A	I	-	Completed	Recurrent malignant glioma (9 GBM, 1 gliosarcoma, 3 AA)	Single IT inj	2 × 10^9^, 2 × 10^10^, 2 × 10^11^, 2 × 10^12^ vp	16.8 *	10.1–47.6 *	Seizure, hemiparesis, thrombocytopenia, hyponatremia, confusion, lethargy	Trask et al. (2000) [24]
AdV-tk + VCV	NCT00589875	IIa	10 January 2008–11 April 2017	Completed	Malignant glioma (34 GBM, 2 AA/AO)	Resection Bed	3 × 10^11^ vp	16.7 *^,§^	Not given	Fatigue, fever, headache, wound complication, seizure	Wheeler et al. (2016) [30]
AdV-tk + VCV	NCT00634231	I	12 March 2008–2 November 2021	Completed	Pediatric malignant glioma (6 GBM, 1 AA, 1 recurrent ependymoma)	Resection Bed	1 × 10^11^, 3 × 10^11^ vp	15.9 *	7.4–37.3 *	Fever, fatigue, nausea/vomiting, hyponatremia	Kieran et al. (2019) [31]
AdV-tk + VCV	NCT00751270	Ib	11 September 2008–4 March 2016	Completed	Malignant glioma (10 GBM, 2 AA)	Resection Bed	3 × 10^10^, 1 × 10^11^, 3 × 10^11^ vp	10.9 *	2.0–46.4 *	Fever, wound complication, nausea/vomiting, transaminitis, hyponatremia, confusion, headache	Chiocca et al. (2011) [32]
AdV-tk + GCV	NCT00870181	II	27 March 2009–25 June 2013	Completed	Recurrent high-grade glioma (14 recurrent GBM, 8 AA/AO)	Intra-arterial cerebral infusion	Not given	10.4 *	2.1–54.9 *	Nausea/vomiting, vasospasm, transaminitis	Ji et al. (2016) [33]
AdV-tk + VCV	NCT03576612	I	3 July 2018–present	Active, not recruiting	Malignant glioma (36 patients allotted to study)	Resection Bed	Not given	-	-	-	-
Ad-RTS-hIL-12 + VDX	NCT02026271	I	1 January 2014–22 September 2021	Completed	Recurrent/progressive GBM or grade III malignant glioma (28 GBM, 3 astrocytoma)	Resection bed inj	2 × 10^11^ vp; 10, 20, 30, or 40 mg VDX	12.7 (20 mg VDX arm)	Not given-30	Lymphopenia, transaminitis, thrombocytopenia, hyponatremia, CRS, headache, confusion, aseptic meningitis	Chiocca et al. (2019) [34]
Ad-RTS-hIL-12 + VDX + Nivolumab	NCT03636477	I	17 August 2018–4 October 2021	Completed	Recurrent or progressive GBM (21 patients enrolled)	Resection bed inj	2 × 10^11^ vp; 10 or 20 mg VDX; 1 or 3 mg/kg nivolumab	9.8	1–24	Transaminitis, brain edema, cold type headache, lymphopenia, CRS	Chiocca et al. (2022) [35]
Ad-RTS-hIL-12 + VDX	NCT03679754	I	20 September 2018–22 September 2021	Completed	Recurrent or progressive GBM (36 patients enrolled)	Resection bed inj	2 × 10^11^ vp; 20 mg VDX	16.2 (unifocal, ≤20 mg dex, *n* = 20)	Not published	Not published	(abstract) Lukas et al. (2020) [36]
Ad-RTS-hIL-12 + VDX + Cemiplimab-rwlc	NCT04006119	II	2 July 2019–11 November 2021	Completed	Recurrent or progressive GBM (40 patients allotted to study)	Resection bed inj	2 × 10^11^ vp; 20 mg VDX; 350 mg cemiplimab-rwlc	Not published	Not published	Not published	(abstract) Lukas et al. (2021) [37]
DNX-2401	NCT00805376	I	9 December 2008–16 July 2018	Completed	Recurrent malignant glioma (33 GBM, 2 AA, 2 gliosarcoma)	Single IT inj (Arm A), Single IT inj + resection bed inj (Arm B)	1 × 10^7^, 3 × 10^7^, 1 × 10^8^, 3 × 10^8^, 1 × 10^9^, 3 × 10^9^, 1 × 10^10^, 3 × 10^10^ vp	9.8 *	2.3–57.9 *	Headache, speech disorder, hemiparesis, convulsion, muscular weakness, visual field defect	Lang et al. (2018) [38]
DNX-2401	NCT01582516	I/II	20 April 2012–9 March 2015	Completed	Recurrent GBM (20 patients allotted)	CED intra- and peritumorally	1 × 10^7^, 1 × 10^8^, 1 × 10^9^, 1 × 10^10^, 3 × 10^10^, 1 × 10^11^ vp	-	-	-	-
DNX-2401 + TMZ	NCT01956734	I	8 October 2013–24 October 2017	Completed	First recurrent GBM (31 patients allotted)	IT or resection bed inj	3 × 10^10^ vp; 150 mg/m^2^ TMZ	-	-	-	-
DNX-2401 + IFN-γ	NCT02197169	Ib	22 July 2014–16 July 2018	Completed	Recurrent GBM or gliosarcoma (27 GB)	Single IT inj	3 × 10^10^ vp; 50 mcg/m^2^ s.c. IFN-γ	Not published (OS 12 m of 33%, 18 m 22%)	Not published	Fatigue, headache, seizures	(abstract) Lang et al. (2017) [39]
DNX-2401 + Pembrolizumab	NCT02798406	II	14 June 2016–15 July 2021	Completed	Recurrent GBM or gliosarcoma (49 patients allotted)	Single IT inj	5 × 10^8^, 5 × 10^9^, 5 × 10^10^ vp; 200 mg pembrolizumab	-	-	-	-
DNX-2401	-	I	-	Completed	Recurrent GBM (19 patients treated)	CED	1 × 10^7^, 1 × 10^8^, 1 × 10^9^, 1 × 10^10^, 3 × 10^10^ vp	4.2	2.2–91.8	Confusion, seizure, increased ICP, meningitis, hydrocephalus	van Putten et al. (2022) [40]
MSC loaded with DNX-2401	NCT03896568	I	1 April 2019–present	Active, recruiting	Recurrent HGG (36 patients allotted)	Intra-arterial	Not given	-	-	-	-
NSC loaded with CRAd-Survivin-pk7	NCT03072134	I	7 March 2017–20 January 2023	Completed	Newly diagnosed malignant glioma (11 GBM, 1 AA)	Resection bed inj	6.25 × 10^10^ vp in 5 × 10^7^ NSCs, 1.25 × 10^11^ vp in 1 × 10^8^ NSCs, 1.875 × 10^11^ in 1.5 × 10^8^ NSCs	18.4	Not published	Meningitis, thromboembolic event, encephalopathy, cerebral edema, muscle weakness	Fares et al. (2021) [41]
NSC loaded with CRAd-Survivin-pk7	NCT05139056	I	1 December 2021–present	Active, recruiting	Recurrent HGG (36 patients allotted)	Intracavitary post resection	Not given	-	-	-	-
M032-HSV-1	NCT02062827	I	14 February 2014–present	Active, not recruiting	Recurrent or progressive GBM, AA, or gliosarcoma (24 patients allotted)	IT catheter infusion	1 × 10^5^, 1 × 10^6^, 1 × 10^7^, 1 × 10^8^, 1 × 10^9^ pfu	-	-	-	-
M032-HSV-1 + Pembrolizumab	NCT05084430	I/II	19 October 2021–present	Active, recruiting	Recurrent, progressive, or newly diagnosed GBM, AA, or gliosarcoma (28 patients allotted)	Not given	Not given; 200 mg pembrolizumab	-	-	-	-
rQNestin34.5v.2 (CAN-3110)	NCT03152318	I	15 May 2017–11 January 2023	Active, recruiting	Recurrent malignant glioma (26 GBM, 1 AA, 3 AO)	Single IT inj	1 × 10^6^ at half-log increments up 1 × 10^10^ pfu	13.25	Not published	Not published	(abstract) Chiocca et al. (2021) [42]
G47∆	UMIN000002661	I/II	23 October 2009–14 March 2019	Completed	Recurrent GBM (13 patients)	IT inj, 2 doses	3 × 10^8^ or 1 × 10^9^ pfu per dose	30.5 (7.3 ^ǂ^)	3.2–143.9 ^ǂ^	Headache, fever, vomiting, leukopenia, CN disorder, seizure	Todo et al. (2022) [43]
G47∆	UMIN000015995	II	18 December 2014–26 June 2020	Completed	Residual or recurrent GBM (19 patients)	IT inj up to 6 doses	1 × 10^9^ pfu per dose	28.8 (20.2 ^ǂ^)	4.2–65.3 ^ǂ^	Fever, vomiting, nausea, lymphocytopenia, leukopenia	Todo et al. (2022) [44]
PVSRIPO	NCT01491893	I	14 December 2011–28 September 2018	Completed	Recurrent malignant glioma (61 GBM)	IT CED	1 × 10^7^, 5 × 10^7^, 1 × 10^8^, 3.3 × 10^8^, 1 × 10^9^, 3.3 × 10^9^, 1 × 10^10^ TCID_50_	12.5	3.1–70.4	Fatigue, gait disturbance, confusion, dysphagia, headache, paresthesia, pyramidal tract syndrome, seizure	Desjardins et al. (2018) [45]
PVSRIPO	NCT02986178	II	8 December 2016–present	Active, not recruiting	Recurrent malignant glioma (122 patients allotted)	IT CED	-	-	-	-	-
PVSRIPO	NCT03043391	Ib	6 February 2017–present	Active, not recruiting	Recurrent malignant glioma, pediatric (12 patients allotted)	IT CED	-	-	-	-	-
PVSRIPO + pembrolizumab	NCT04479241	II	21 July 2020–present	Active, not recruiting	Recurrent GBM (30 patients allotted)	IT CED	-	-	-	-	-
PVSRIPO	NCT04599647	Expanded access	23 October 2020–29 June 2022	No longer available	N/A	IT CED	5 × 10^7^ TCID_50_	-	-	-	-
Toca 511 + Toca FC	NCT01156584	I	5 July 2010–21 May 2018	Completed	Recurrent HGG (36 patients at time of abstract, subtypes not published)	IT inj (24 patients) or CED (12 patients), Toca FC oral	3.9 × 10^6^ TU, half logs to a maximum of 1.5 × 10^9^ TU; 120 mg/kg/day or 300 mg/kg/day Toca FC	Not published	Not published	-	(abstract) Aghi et al. (2014) [46]
Toca 511 + Toca FC	NCT01470794	I	11 November 2011–21 May 2018	Completed	Recurrent HGG (46 GBM, 6 AA, 4 other)	Resection bed inj, Toca FC oral	Not given	11.9	Not published	Rash, mucositis, facial swelling, hemorrhagic enteritis, colitis, nausea, vomiting, diarrhea	Cloughesy et al. (2018) [47]
Toca 511 + Toca FC	NCT01985256	I	15 November 2013–21 May 2018	Completed	Recurrent HGG (17 patients allotted)	IV + resection bed inj, Toca FC oral	4.6 × 10^9^ TU IV/day for 3 days, 9.5 × 10^9^ TU IV/day for 5 days. (1.2 × 10^9^ TU inj tumor bed); 220 mg/kg/day Toca FC	Not published	Not published	Not published	(abstract) Cloughesy et al. (2015) [48]
Toca 511 + Toca FC	NCT02414165	II/III	10 April 2015–7 February 2020	Terminated	Recurrent GBM or AA (171 GBM, 30 AA)	Resection bed inj, Toca FC oral	4 × 10^8^ TU; Toca FC 220 mg/kg/d	11.1	Not published	Aphasia, hemiparesis, headache, seizure	Cloughesy et al. (2020) [49]
Toca 511 + Toca FC	NCT02598011	Ib	5 November 2015–30 March 2020	Withdrawn	Recurrent HGG	Resection bed inj, Toca FC oral	-	-	-	-	-
Toca 511 + Toca FC	NCT04105374	II/III	26 September 2019–24 March 2020	Withdrawn	Newly diagnosed GBM	Intracranial injection	-	-	-	-	-

## Data Availability

Not applicable.
